# Comprehensive Comparative Analysis of the GATA Transcription Factors in Four Rosaceae Species and Phytohormonal Response in Chinese Pear (*Pyrus bretschneideri*) Fruit

**DOI:** 10.3390/ijms222212492

**Published:** 2021-11-19

**Authors:** Muhammad Aamir Manzoor, Irfan Ali Sabir, Iftikhar Hussain Shah, Han Wang, Zhao Yu, Faiz Rasool, Muhammad Zaid Mazhar, Shoaib Younas, Muhammad Abdullah, Yongping Cai

**Affiliations:** 1 School of Life Sciences, Anhui Agricultural University, Hefei 230036, China; aamirmanzoor1@hotmail.com (M.A.M.); hwang_0707@163.com (H.W.); zyannong@stu.ahau.edu.cn (Z.Y.); 2 School of Agriculture and Biology, Shanghai Jiao Tong University, Shanghai 200240, China; irfanalisabir@sjtu.edu.cn (I.A.S.); iftikharhussainqau@gmail.com (I.H.S.); 3 Gulab Davi Education Institute, Lahore 200240, Pakistan; mudassarraj123@gmail.com; 4 Department of Agriculture, University of Agriculture, Faisalabad 38000, Pakistan; xgujjar67@gmail.com; 5 Department of Food Science and Technology, University of Central Punjab, Lahore 200240, Pakistan; shoaibfsn@yahoo.com; 6 Queenland Alliance of Agriculture and Food Innovation, The University of Queensland, Brisbane 4072, Australia; abdullahpadana@hotmail.com

**Keywords:** GATA transcription factor, evolution, phylogenetic analysis, subcellular localization, abiotic stress, gene expression

## Abstract

The GATA gene family is one of the most important transcription factors (TFs). It extensively exists in plants, contributes to diverse biological processes such as the development process, and responds to environmental stress. Although the GATA gene family has been comprehensively and systematically studied in many species, less is known about GATA genes in Chinese pears (*Pyrus bretschneideri*). In the current study, the GATA gene family in the four Rosaceae genomes was identified, its structural characteristics identified, and a comparative analysis of its properties was carried out. Ninety-two encoded GATA proteins were authenticated in the four Rosaceae genomes (*Pyrus bretschneideri*, *Prunus avium*, *Prunus mume*, and *Prunus persica*) and categorized into four subfamilies (Ⅰ–Ⅳ) according to phylogeny. The majority of GATA genes contained one to two introns and conserved motif composition analysis revealed their functional divergence. Whole-genome duplications (WGDs) and dispersed duplication (DSD) played a key role in the expansion of the GATA gene family. The microarray indicated that, among *P. bretschneideri*, *P. avium*, *P. mume* and *P. persica*, GATA duplicated regions were more conserved between *Pyrus bretschneideri* and *Prunus persica* with 32 orthologous genes pairs. The physicochemical parameters, duplication patterns, non-synonymous (ka), and synonymous mutation rate (ks) and GO annotation ontology were performed using different bioinformatics tools. *cis*-elements respond to various phytohormones, abiotic/biotic stress, and light-responsive were found in the promoter regions of GATA genes which were induced via stimuli. Furthermore, subcellular localization of the *PbGATA22* gene product was investigated, showing that it was present in the nucleus of tobacco (*Nicotiana tabacum*) epidermal cells. Finally, in silico analysis was performed on various organs (bud, leaf, stem, ovary, petal, and sepal) and different developmental stages of fruit. Subsequently, the expression profiles of *PbGATA* genes were extensively expressed under exogenous hormonal treatments of SA (salicylic acid), MeJA (methyl jasmonate), and ABA (abscisic acid) indicating that play important role in hormone signaling pathways. A comprehensive analysis of GATA transcription factors was performed through systematic biological approaches and comparative genomics to establish a theoretical base for further structural and functional investigations in Rosaceae species.

## 1. Introduction

Transcription factors (TFs) are a major component that regulates gene expression by binding to the gene’s promoter and controlling various biological processes. The capacity of TFs to bind *cis*-acting elements on the promoter regions can be used to classify them into various categories [1]. Based on the existence of DNA binding motifs, these transcription factors have been assigned the names zinc-finger, MYB, WRKY, PHD (plant homeodomain), AP2/EREBP (Apetala2/ethylene-responsive element-binding protein), MADS, bZIP (basic leucine zipper), and NAC (NAM, ATAF1/2, and CUC1/2) [2,3,4]. The zinc-finger transcription factors are divided into different families related to their conserved domain structure [5]. GATA transcription factors are distinguished by their ability to bind the W-G-A-TA-R (W = T/A, R = G/A) sequences on the promoter regions [6]. Type IV zinc-finger motifs with the CX2CX1720CX2C consensus sequence is accompanied by a specific region that facilitates DNA binding. The members of GATA zinc-finger (with 17–18 residues in the binding loop) are present in both animal and fungal GATA TFs, while 17–20 residues are present in plants’ GATA TFs [7]. The function of GATA transcription factors in animals and fungi has been extensively investigated [8,9,10] while in plants, GATA transcription factors are directly implicated in the regulation of stress signaling and metabolic pathways in addition to their active role in cell differentiation [11,12]. In terms of the amino acid residues available in the zinc-finger loop, fungal GATA transcription factors are a composite of plant and animal GATA transcription factors. GATA transcription factors execute several functions in fungal cells, including siderophore formation, circadian regulation, and nitrogen metabolism [13,14]. GATA transcription factors have still not been thoroughly investigated in plants. NTL1 is a homolog of NIT-2, the first plant GATA transcription factor discovered in tobacco which plays a key role in nitrogen metabolism [15]. The GATA TFs are associated with the regulation of various stress-sensitive genes, light response, floral development-related genes, and hormonal signaling such as cytokinin, auxin, and gibberellin in plants [7,16,17]. Besides that, in Arabidopsis, the active function of GATA TFs in photooxidative damage prevention through tetrapyrrole biosynthesis (TPB) has been quite well reported [18]. GNL-class B GATA (GNC-LIKE) TFs were found to function downregulated for type-B ARRs in Arabidopsis and were thus coordinated at the junction of cytokinin signaling and auxin [19]. Photomorphogenesis is regulated by GATA2 TFs, which is a key role in light signaling, while CGA1 and GNC are the main transcriptional regulators and are involved in chloroplast biogenesis in Arabidopsis [17,20]. In addition, hormonal signals, such as auxin and gibberellin regulation, are governed by the GATA factor and control the downregulation of target genes GNL and GNC during plant development/growth. These stimuli also manipulate the brassinosteroid level and facilitates the regulation of the GATA2 (which is a transcription factor of GATA in Arabidopsis) [20,21]. Studies have endeavored to investigate the role of GATAs in stress response and hormonal signaling, especially gibberellin, auxin, and jasmonic acid signaling, but there have been limited publications regarding this subject.

Due to changes in the environment, abiotic stress is a vital element that affects the final yield of fruit crops by manipulating fruit growth and development [22,23]. In temperate climates, the pear (*Pyrus bretschneideri*) is cultivated to produce fruit with high commercial value [24,25]. For sustainable agriculture, investigating the mechanisms of pear fruit growth and development, as well as abiotic stress tolerance, is critical. Although the importance of GATA TFs in model plants such as Arabidopsis and rice has been studied, the characterization of the pear GATA family and its response to abiotic stress and hormonal signaling remains uninvestigated. This study was planned to reveal all aspects of the GATA family for abiotic stress and hormonal signaling.

## 2. Results

### 2.1. Identification and Classification of GATA Gene Family in Four Rosaceae Species

To identify the GATA gene family, we utilized the four species genomes data for BLASTP and Pfam and HMM searches [26,27]. All potential GATA genes were confirmed using plant transcription factors database and Pfam (http://pfam.xfam.org/, accessed on 5 February 2021) [28,29] and SMART (http://smart.embl-heidelberg.de/, accessed on 5 February 2021) to ensure that they included the GATA domains [30]. The protein sequence and the coding sequence were obtained from the database project of Chinese pear and other three species (peach, sweet cherry, and Japanese apricot) from the Genome Database for Rosaceae (GDR). A total of 92 GATA members were investigated from four Rosaceae species with 32 from *Pyrus bretschneideri* (*PbGATA1-PbGATA32*), 18 from *Prunus avium* (*PaGATA1-PaGATA18*), 20 proteins from *Prunus mume* (*PmGATA1-PmGATA20*), and 22 from *Prunus persica* (*PpGATA1-PpGATA22*).

**Figure 1:**
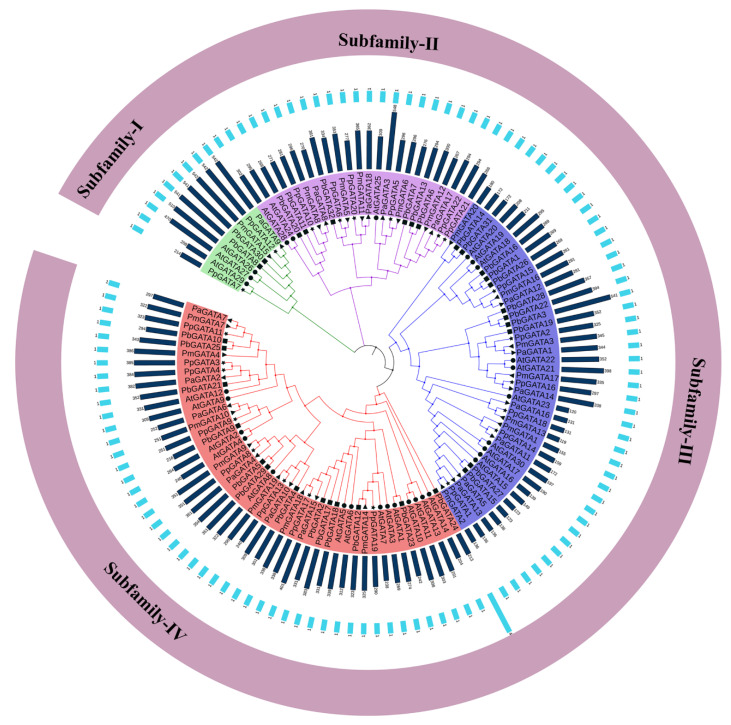
Phylogeny analyses of GATA gene family in the Rosaceae species. The phylogenetic tree 127 of GATA genes was obtained using the protein sequences by maximum likelihood-method with 1000 bootstrap replicates. The branches of the mixed clade built-in four Rosaceae species (*Prunus avium*, *Prunus mume*, *Pyrus bretschneideri,* and *Prunus persica*) with Arabidopsis and different colors indicating the GATA gene classification. The blue bars and light blue bars indicate the number of amino acids and the number of domains, respectively.

Moreover, the evolutionary relationship was investigated between four Rosaceae species (*Pyrus bretschneideri*, *Prunus avium*, *Prunus mume*, and *Prunus persica*). We retrieved 30 GATA protein sequences from the Arabidopsis genome [31] to further identify and reveal the potential evolutionary history of GATA genes. All GATA genes were aligned through Clustalx software for the construction of a phylogenetic tree (Figure 1). The maximum likelihood method (ML-M) was utilized with 1000 times bootstrap and other default parameters for phylogeny. 30 *Arabidopsis thaliana* GATA genes along with 30 *PbGATA*, 20 *PmGATA*, 18 *PaGATA*, and 22 *PpGATA* genes were classified into four subfamilies (I to IV). According to the phylogeny, subfamily- I contained 9 while subfamily- II, III, IV comprised 24, 41, and 48 genes, respectively. These findings indicated evolutionary conservation and a stronger homology between GATA genes in highly associated subfamily whenever evaluated simultaneously. The length of GATA proteins varied from 119 (*PmGATA13*) to 548 (*PaGATA3*) with an average of 300.2 amino acids in four Rosaceae species, as shown in Table S1. GATAs protein molecular weights and isoelectric points (pI) were anticipated to be between 12,985.2 and 60,234.7 kDa with an average of 32502.5 kDa and between 4.7 and 10.0 (with an average of 10.01), respectively (Table S1). At least one domain was identified in all members of four Rosaceae species (Figure 1). Current findings reveal that the 92 predicted proteins have varying pI and molecular weights due to their various protein lengths. It was suggested that various GATA proteins might have a functional divergence.

### 2.2. Gene Structure and Conserved Motifs of GATA Gene Family

**Figure 2:**
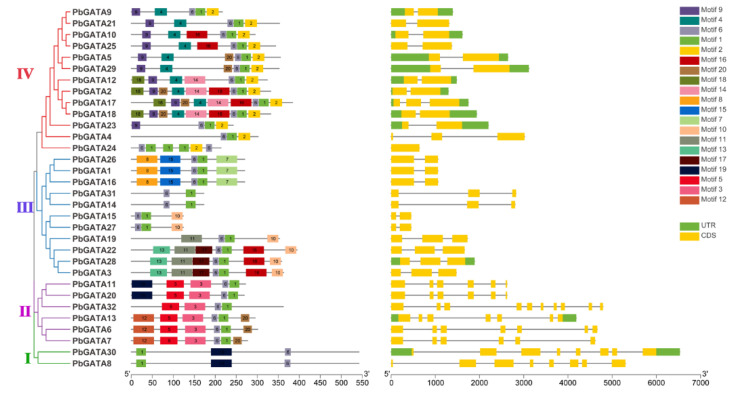
Phylogenetic tree, conserved motif, and gene structure analysis of GATA genes. Phylogenetic relationship (**left**); 20 conserved motifs analysis was performed using MEME (motif-based sequence analysis tool) and each motif is represented with a colored box (**middle**). The introns-exons organization in GATA genes is denoted; yellow boxes represent exons, introns are represented with thin black lines, and the green boxes indicated the untranslated (UTR) region (**right**).

The conserved motif analysis of the *PbGATA* family was investigated to validate their evolutionary relationship and classification. The MEME program [32] was used to predict and validate the conserved motifs in the *PbGATA* proteins to learn more about their sequence features (Figure 2). The conserved motifs (1 and 9) were identified across all GATA proteins. From the findings, the majority of *PbGATA* members in the same group have the same pattern. The highest conserved motifs (9) were observed in subfamily-IV, while subfamily-I contained the lowest conserved motifs (3). Some proteins, on the other hand, contain many distinct conserved motifs in the various subfamily. For example, motifs 1 and 6 were found only in subfamilies (I, II, III, and IV). The same motif in all subfamilies revealed that they might be necessary for some basic functions.

Genetic variation is a vital component for the evolution of various gene families. To further describe and comprehend the structural diversity of the GATA gene, we conducted an exon-intron investigation. The quantity of introns/exons varies from 1–9/1–10 in subfamily II; *PbGATA32* had the most (9/10), while in subfamily III, *PbGATA*15,27 had the least introns and exons (1/2) (Figure 2 and Table S1). The number of introns ranged from 0 to 9, indicating a wide range of variability. Furthermore, the *PbGATA* genes which were classified together in the same subfamily had extremely identical exon-intron structures, verifying a close evolutionary connection and group categorization. It was also confirmed that the length of genes differed in every subfamily. The evolutionary relationship of the *PbGATA* gene family was predicted, which led to functional variations among GATA genes in Chinese pear.

### 2.3. GO Annotation Analysis

**Figure 3:**
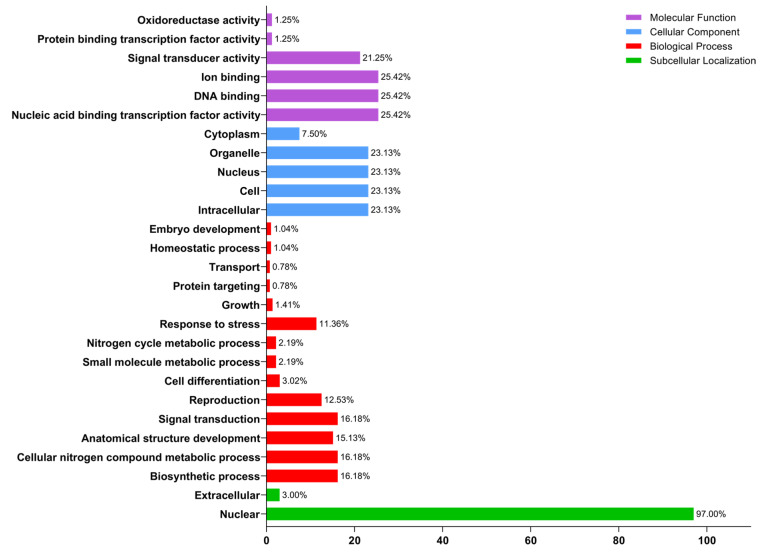
GO annotations enrichment analysis for GATA proteins. The GO annotation is divided into four main categories: molecular function, cellular component, biological process, and subcellular localization. The y-axis on the right represents the percentage of genes in each category and the left showed the Go target functions.

Subcellular location and the functions of the potential GATA protein are predicted while using (GO) gene ontology annotation analysis in pear. 32 GATA proteins were classified into 27 functional groups based on amino acid resemblances and divided into four ontologies, namely molecular function, cellular component, biological process, and subcellular localization (Table S3). In subcellular localization, we examined that 97% annotated GATA protein anticipated their function into nuclear followed by extracellular 3%. In molecular function annotation, GATA protein anticipated their maximum functionality (25.42%) in ion binding, DNA binding, and nucleic acid binding transcription factor, followed by signal transducer activity (21.25%), protein binding transcription factor (1.25%), and oxidoreductase activity (1.25%). In cellular component annotation, *PbGATA* protein annotated with intracellular, cell, cytoplasm, nucleus, an organelle with 23.13% followed by cytoplasm (7.50%). Moreover, predicted GATA proteins were annotated with signal transduction, cellular nitrogen compound metabolic process, and biosynthetic process along with the same percentage (16.18%) while a response to stress, reproduction, and cell differentiation contribute 11.36%, 12.53%, and 3.02%, respectively (Figure 3).

### 2.4. Chromosomal Distribution and Gene Duplication Events and Ka/Ks Analysis 

We visualized a map of chromosomal locations based on the genomic data of Chinese pear, sweet cherry, peach, and Japanese apricot. In Chinese pear, 78.1% of genes were distributed on the chromosome, while 21.9% were traced on the unassembled scaffold. Moreover, maximum *PbGATA* genes (7) were identified on chr15 while, chr6,7,11,13 and 17 contain at least 1 gene number. Subsequently, in sweet cherry and Japanese apricot, all genes were traced on chromosomes which were arranged in clusters formation. Meanwhile, 90% of *PmGATA* genes were noticed on chromosomes, remaining 10% were scattered on the unassembled scaffold. The maximum number of *PmGATA* genes (4) were visualized on chr4 and chr6, 4 *PaGATA* genes on chr7, and 7 *PpGATA* genes on chr1. (Table S5).

**Figure 4:**
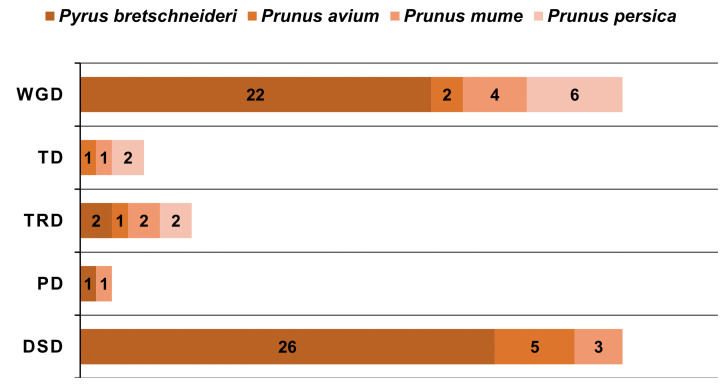
Mode and the total number of gene duplications (DSD, dispersed duplicates; PD, proximal duplicates; WGD, whole-genome duplicates; TRD, transposed duplicates; TD, tandem duplicates) of GATA gene family in Rosaceae species. Different bars indicate the duplicated gene pairs, and each color represents each species.

To further comprehend the extensive mode of gene duplications were identified in Chinese pear, sweet cherry, Japanese apricot, and peach. We investigated five modes of gene duplications in all GATA genes families, including proximal duplication (PD), tandem duplication (TD), dispersed duplication (DSD), transposed duplication (TRD), and whole-genome duplication (WGD) in order to give more relevant information regarding duplication patterns and evolutionary connections amongst these genes (Figure 4).

**Figure 5:**
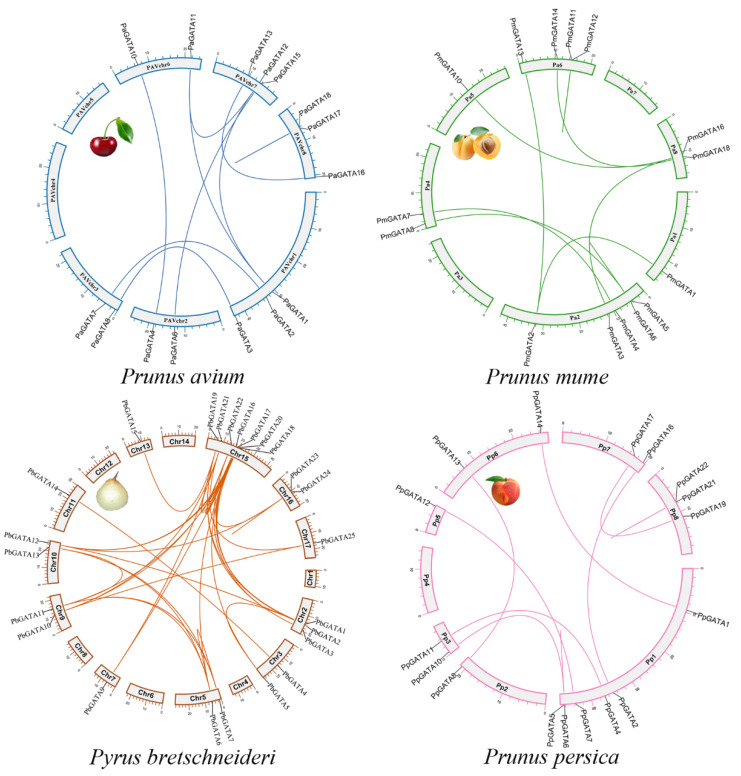
Gene duplication and chromosomal localization of *Prunus avium*, *Prunus mume*, *Prunus persica*, and *Pyrus bretschneideri.* Duplicated gene pairs are linked with a colored line.

WGD and TRD duplication events were found in all four species while DSD duplication event was traced only in pear, sweet cherry, and Japanese apricot. TD duplication was only noticed in peach, Japanese apricot, and sweet cherry. Surprisingly, 81 duplicated gene pairs were traced in four Rosaceae genomes, with a maximum number of duplicated gene pairs derived from DSD (dispersed duplications) (26 pairs out of 81), followed by WGD (whole-genome duplications) (22 pairs out of 81) indicating that the expansion of the GATA genes was linked with DSD and WGD duplication. On the other hand, *P. bretschneideri* 32% and 27% GATA genes had to contribute in DSD and WGD (Figure 5).

**Figure 6:**
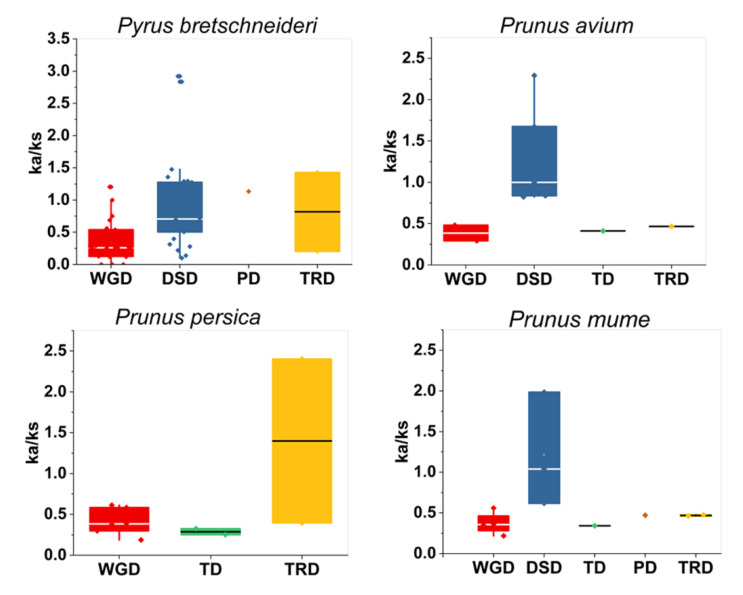
Ka/Ks values of GATA genes family in four Rosaceae species. Comparison of Ka/Ks values for different modes of gene duplications. DSD, dispersed duplicates; PD, proximal duplicates; WGD, whole-genome duplicates; TRD, transposed duplicates; TD, tandem duplicates.

These data validates the close evolution history of these four species. Moreover, these duplication events were also responsible for the expansion of the GATA family in four Rosaceae species. The nonsynonymous (ka) and synonymous (ks) values are used to estimate evolutionary history and gene selection pressures [33]. Amongst these GATA genes, we computed the frequency of nonsynonymous/ synonymous substitutions (ka/ks) in four Rosaceae species. Positive selection was indicated by ka/ks > 1, whereas negative selection with functional limitations were indicated by ka/ks < 1. The mean ka/ks value of whole-genome duplication (WGD) events in *P. persica*, *P. bretschneideri*, *P. avium*, and *P. mume* were 0.40, 0.37, 0.38, 0.37 correspondingly (Table S5). The ka/ks ratio of duplicated gene pairs in *P. avium, P. mume*, *P. bretschneideri*, and *P. persica* were <1, suggesting that GATA genes had a strong purifying selection. However, strawberry, Japanese apricot, Chinese pear, peach, and sweet cherry duplicated gene pairs had higher ka/ks ratio, demonstrating that the GATA family expansion has a complex evolutionary history (Figure 6 and Table S5).

### 2.5. Collinearity Relationships

**Figure 7:**
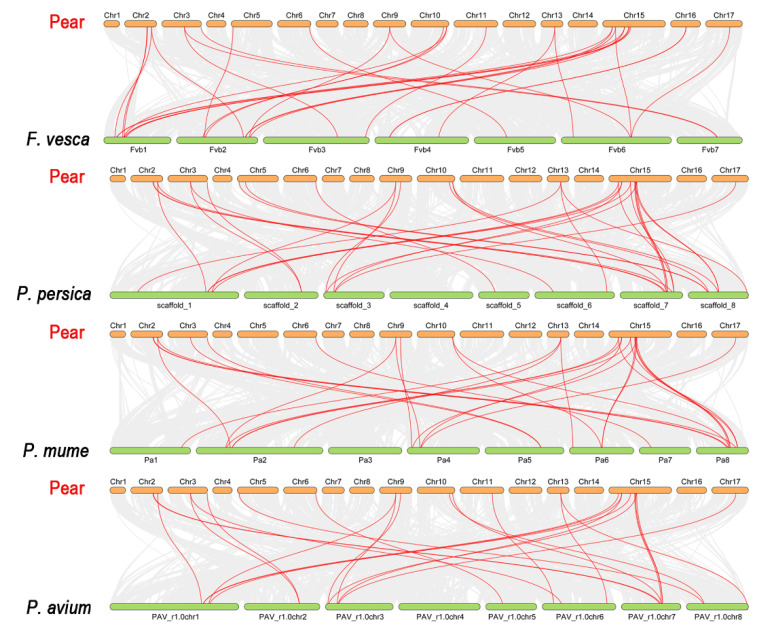
Collinearity analysis of GATA genes family in *Pyrus bretschneideri* between *Fragaria vesca*, *Prunus persica*, *Prunus mume*, and *Prunus avium.* The red line represents the collinearity relationship among Rosaceae species.

The collinearity interactions of GATA genes were studied among *Fragaria vesca*, *P. avium*, *P. mume*, *P. persica*, and *P. bretschneideri* as these five species belong to the Rosaceae family and had a common ancient (Table S4). Total 114 collinear gene pair were traced among the five Rosaceae species, containing 32 orthologous gene pairs among Chinese pear and peach, 29 orthologous genes pairs between pear and strawberry, 25 orthologous gene pairs between sweet cherry and Chinese pear, and 28 orthologous gene pairs among Chinese pear and Japanese apricot, indicating a very close association between four Rosaceae species (Figure 7).

### 2.6. Activity Analysis of PbGATA Promoter

**Figure 8:**
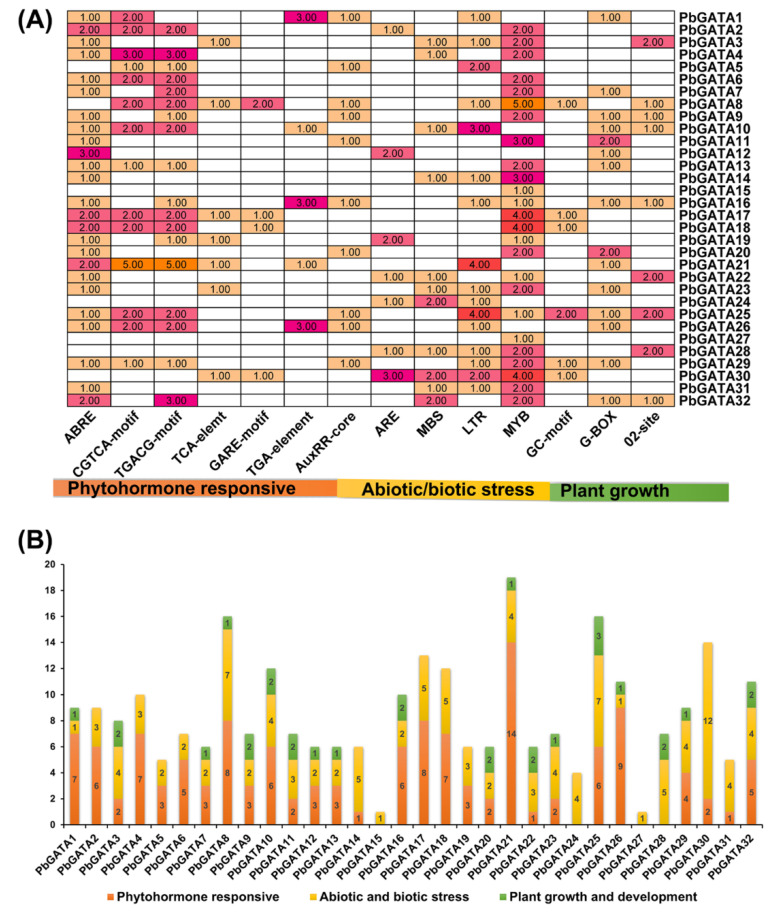
Promotor *cis*-acting elements of GATA family in *Pyrus bretschneideri*. (**A**) The *cis*-elements on the upstream region of GATA genes with functional similarity are indicated by a specific different coding color. (**B**) *cis-*elements of each category (phytohormones responsive, abiotic/biotic stress, and plant growth development) represent different colors.

We used the PlantCARE database to find probable *cis*-acting elements to better understand the *PbGATA* gene expression regulation mechanism (Table S6). These *cis*-acting elements were primarily classified into three physiological processes, containing plant growth/development, phytohormones, and biotic/abiotic stress-responsive. Most of the GATA gene family contained *cis*-acting elements: ABRE, CGTCA-motif, TGACG-motif, TCA-element, GARE-motif, TGA-elements, AuxRR-core, ARE, MBS, LTR, MYB, GC-motif, G-box, and 02-site (Figure 8).

Many stress-related *cis*-elements were examined ARE, MBSs, LTRs, MYB, and GC-motif, which are connected to anaerobic induction, drought initiation, salt/ drought stress-responsive, various development process/stress, and involved in anoxia [34,35]. Phytohormones related to *cis*-elements like the TGACG-motif, ABRE, TCA-element, AuxRR-core, CGTCA-motif, GARE-motif, and TGA-elements were also identified and have been related with SA, auxin, ABA, MeJA and gibberellin responses, respectively (Figure 8 and Table S6).

In the growth/development group, *cis*-acting elements were placed widely throughout the promoter regions, including O2-site (involved in zein metabolism regulations) and G-Box-4 (responsible for plant growth in response to light). G-Box-4 covered the largest portion (57%) and O2-site (43%) (Figure 8A,B). These findings indicate that *PbGATAs* have the potential to respond to phytohormones (ABA, SA, MeJA, and auxin) and improve abiotic/biotic stress.

### 2.7. Transcriptomic Pattern of the PbGATA Genes

To explore expression profiles of *PbGATA* genes were investigated through transcriptomic data in different tissues (sepal, bud, leaf, ovary, and petal) and different development stages of fruit (stage1-15DAB, stage2-30DAB, stage3-55DAB, stage4-85DAB, stage5-115DAB, stage6- mature stage, and stage7-fruit senescence stage). Fragments per kilobase million (FPKM) values were utilized to examine the gene expression.

**Figure 9:**
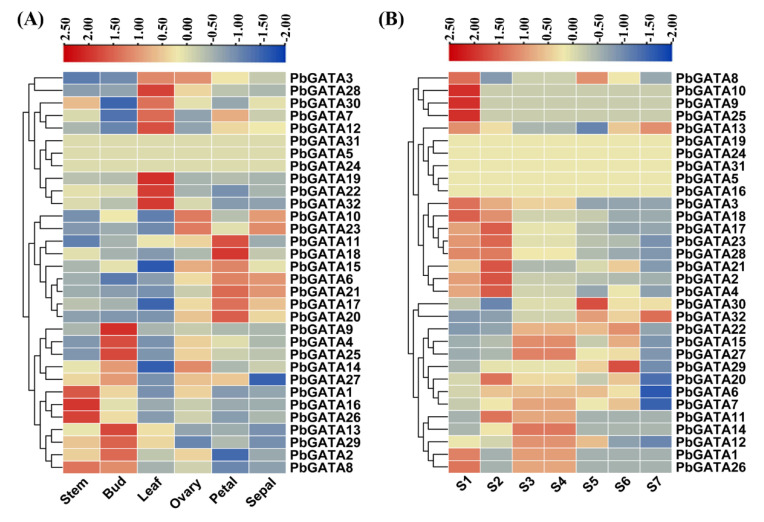
A heat map is visualized to demonstrate the relative expression patterns of GATA genes. (**A**) The transcription patterns of the GATA gene family in organs (leaf, ovary, sepals, petals, bud, and stem). (**B**) The fruit of Chinese pear at different developmental stages (S1 to S7). Different colors correspond to log2 transformed values. Blue or violet color indicates no or lower relative transcripts abundance of each sample and red showed higher expression respectively.

In this study, *PbGATA* genes had stage-specific expression. Two genes (*PbGATA26*, *PbGATA16*) in the stem, four genes (*PbGATA13, PbGATA25, PbGATA9, PbGATA4*) in the bud, five genes (*PbGATA32*, *PbGATA22*, *PbGATA19*, *PbGATA12*, *PbGATA28*) in leaf, two genes (*PbGATA18*, *PbGATA11*) in petal, four genes (*PbGATA21*, *PbGATA6*, *PbGATA23*, *PbGATA10*) in sepal and three genes (*PbGATA23*, *PbGATA10*, *PbGATA3*) in the ovary were extremely expressed while no expression was identified in *PbGATA24, PbGATA5,* and *PbGATA31* in any stage (Figure 9A and Table S7). These results demonstrated that GATA genes play a vital role in floral stages. Several genes expressed their peak expression in different fruit development stages such as S1 (*PbGATA10*, *PbGATA9*, *PbGATA25)*, S2 *(PbGATA21*, *PbGATA2*, *PbGATA4*), S5 (*PbGATA30*), and S6 (*PbGATA29*), while moderate expression was noticed in S3 and S4 (*PbGATA14*, *PbGATA27*, *PbGATA15*) and S7 (*PbGATA32* and *PbGATA13*), S1 (*PbGATA*10, *PbGATA*9, *PbGATA*25), S2 (*PbGATA*21, *PbGATA*2, *PbGATA*4) S3 and S4 (*PbGATA*14, *PbGATA*27, *PbGATA*15 moderately express), S5 (*PbGATA*30) S6 (*PbGATA*29) and S7 (*PbGATA*32 and *PbGATA*13 moderately) (Figure 9B and Table S7). Surprisingly, the *PbGATA32* gene gradually increase and showed that this gene had a potential role in a later stage, indicating that might be *PbGATA* genes play an important role in fruit development and ripening (Figure 9A,B).

### 2.8. Subcellular Localization Analysis

**Figure 10:**
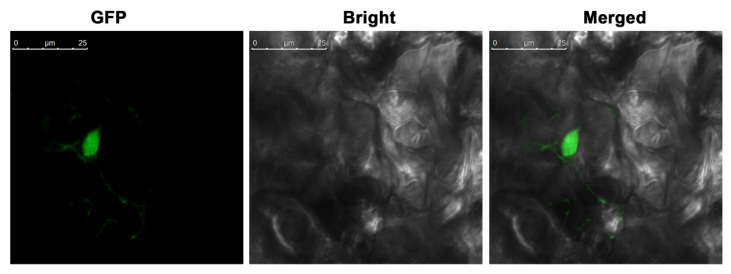
Analysis of *PbGATA22* subcellular localization in tobacco leaf epidermal cells. Green fluorescent protein (GFP) fluorescence is shown in green color, Bars = 25 μm. Confocal images demonstrate the nucleus localization when transiently expressed in the epidermal cells of *N. benthamiana* leaves.

To analyze the subcellular localization, we transiently overexpressed the *PbGATA22* gene fused with eGFP into *Nicotiana benthamiana* leaves through agroinfiltration. In this construct, *PbGATA22* protein was fused to the N-terminus of GFP protein under the control of the CaMV 35S promoter produced a strong green fluorescent signal in the nucleus (Figure 10) which is consistent with the previous results [36]. These results suggested that *PbGATA22* was indeed localized in the nucleus.

### 2.9. Gene Expression Pattern by qRT-PCR of PbGATA after Hormonal Treatment

**Figure 11:**
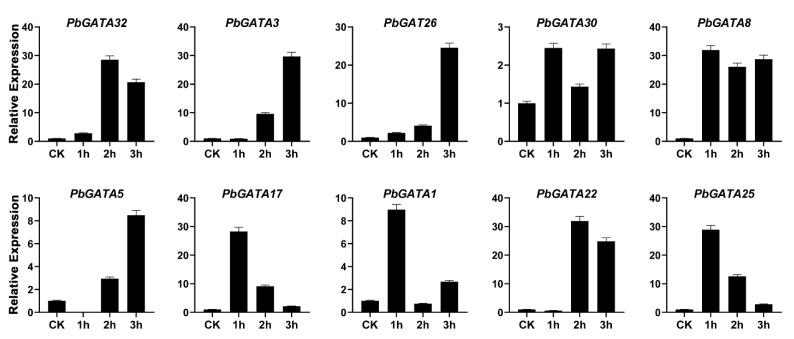
Expression profiling of GATA gene family analyzed by qRT-PCR in the fruit of Chinese pear at the developmental stage after hormonal treatment of abs*cis*ic acid (ABA) and CK represents control. Error bars represent the standard error (SE) of three biological replicates.

To explore the expression profile of the GATA gene family in response to exogenous hormonal treatment. We selected 10 *PbGATAs* members and analyzed expression level using qRT-PCR after foliar application of hormones such as methyl jasmonate (MeJA), salicylic acid (SA), and abs*cis*ic acid (ABA) on pear fruits. In ABA-treated fruits after one hour, the expression of *PbGATA8*, *PbGATA17*, *PbGATA1*, *PbGATA25*, *PbGATA30* were upregulated (31.9, 8.9, 28.9, 2.4-fold, respectively). Furthermore, *PbGATA32* and *PbGATA22* revealed peak expression at 2 h (28.4, 31.9 times, respectively), while *PbGATA3*, *PbGATA26*, and *PbGATA5* (29, 6, 24.5, 28.3-fold respectively) showed the highest expression at 3 h as compared to control (CK) (Figure 11).

**Figure 12:**
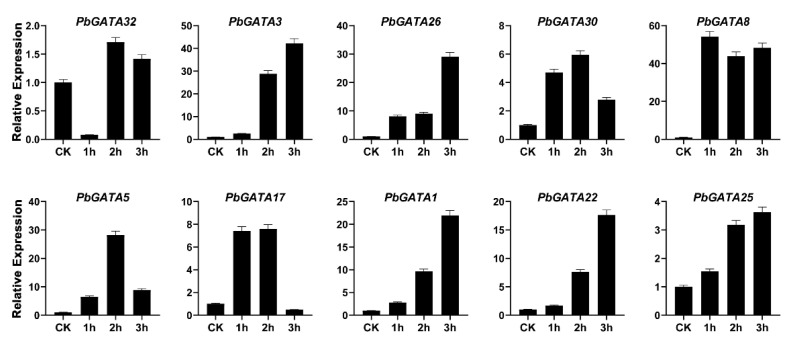
Expression profiling of GATA gene family analyzed by qRT-PCR in the fruit of Chinese pear at the developmental stage after hormonal treatment of salicylic acid (SA) and CK represents control. Error bars represent the standard error (SE) of three biological replicates.

Moreover, after one hour of SA treatment, the expression profile *PbGATA8* upregulated along with 54.2-fold, meanwhile, *PbGATA32*, *PbGATA30*, *PbGATA5*, and *PbGATA17* were highly expressed at 2 h (1.7, 5.9, 28.2, 7.5 times, respectively, compared to control). *PbGATA5*, *PbGATA17*, *PbGATA3*, *PbGATA26*, *PbGATA1*, *PbGATA22* and *PbGATA25* peaked (42.1, 29.1, 21.9, 17.6, and 3.6-fold, respectively) at 3 h (Figure 12).

**Figure 13:**
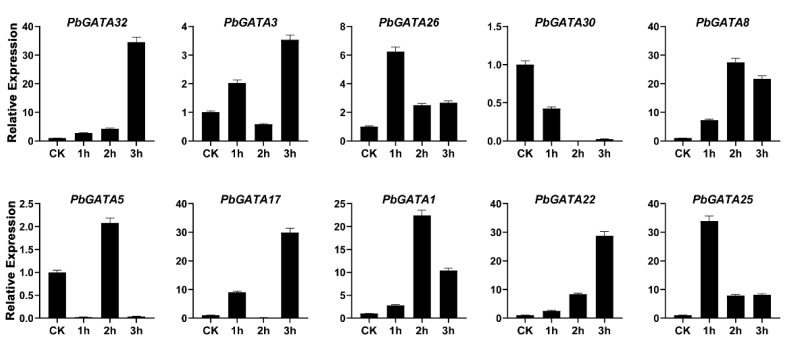
Expression profiling of GATA gene family analyzed by qRT-PCR in the fruit of Chinese pear at the developmental stage after hormonal treatment of methyl jasmonate (MeJA) and CK represents control. Error bars represent the standard error (SE) of three biological replicates.

In the treatment of MeJA, The *PbGATA26* (6.2) and *PbGATA25* (33.9) were upregulated after 1 h after treatment. Furthermore, the expression *PbGATA*22 (28.8), *PbGATA8* (31.9), *PbGATA5* (27.4) peaked at 2 and *PbGATA32*, *PbGATA3*, *PbGATA17*, *PbGATA1*, *PbGATA22* (34.5, 3.5, 29.9,10.4, 28.8 -fold, respectively) peaked at 3 h (Figure 13). *PbGATA30* only showed expression patterns after the SA and ABA treatment but remain silent in MeJA treatment. This result illustrated that the *PbGATA30* function is only related to ABA and SA.

## 3. Discussion

GATA transcription factors (TFs) are involved in various important biochemical and physiological processes in plants [37,38,39,40]. Genome-wide investigation of the GATA gene family was conducted to find expression diversity and potential functions in four Rosaceae species. In the present study, we investigated 92 genes of GATA transcription factors in four Rosaceae spp, namely as *PbGATA*1-30 (*Pyrus bretschneideri*), *Pm1-18* (*Prunus mume*), *Pp1-Pp20* (*Prunus persica*), and *Pa1-Pa18* (*Prunus avium*) based on chromosomal location. Bioinformatics analysis such as phylogeny, conserved motifs, domains, orthologous genes, gene structure, chromosomal location, physiochemical properties, *cis*-elements, and gene duplication events was performed in the GATA gene family. Moreover, RNA-seq, subcellular localization, and qRT-PCR analysis under hormonal treatments were analyzed. These results suggested that GATA genes are classified into four subfamilies (I-IV) according to phylogeny and genetic structure. Subfamily-IV had the highest *PbGATA* genes, which was similar to *A. thaliana* [40].

Additionally, the orthologous syntenic relationship among *Pyrus bretschneideri, Prunus avium*, *Prunus persica*, *Prunus mume*, was analyzed. *Pyrus bretschneideri* and *Prunus avium* contained a maximum (25) gene pairs, followed by *Pyrus bretschneideri* and *Prunus mume* (33 gene pairs), and *Pyrus bretschneideri* and *Prunus persica* (32 gene pairs), while 29 orthologous gene pairs were identified in *Pyrus bretschneideri* and *Fragaria vesca*. These results also revealed that all of the GATA homologous gene pairs were firmly clustered together, suggesting that they were more strongly linked to one another. Syntenic patterns may provide details about the evolution of a genome. Due to chromosomal localizations, fusions, and selective gene loss, certain homologous GATA genes may not have been mappable to any syntenic regions, making chromosomal syntenic difficult to identify (Figure 7 and Table S4) [41]. Taken altogether, our analysis showed that *PbGATA* genes have physicochemical features that are highly conserved across species. Gene duplication events are a vital mechanism for generating various genetic novelty in plants, which could improve the organism’s ability to adapt the environmental stress. The revolution in plant genomes is facilitated by gene duplication events [42,43]. Gene duplication events (segmental, tandem, and whole-genome duplication) are crucial for evolution. Consequently, identifying duplication mode help in the function and the structure of the GATA gene family [40,44]. Our results indicate that whole-genome duplication (WGDs) is more common in Roseacea species as compared to tandem duplication (Figure 4 and Table S5). Many GATA genes in pears have two or more equivalents in *A. thaliana*, suggesting that genome duplication events may have led to the amplification of the *PbGATA* gene family in pears. Furthermore, four and six gene pairs were discovered after further analysis, both of which were thought to have resulted from segmental and whole-genome duplication events, correspondingly. Moreover, the motif seen in all GATA proteins, various *PbGATA* groups included additional conserved motifs. The existence of these diverse, highly conserved domains might therefore be linked to diverse *PbGATA* protein activities. Exon gain/loss has occurred often during the history of numerous gene families. Most GATA genes in group A in *A. thaliana* have just two exons. On the other hand, *PbGATA* has one exon/intron and *PbGATA32* contained the highest introns and exons (Figure 2B). Taken together, our findings show that GATA genes have experienced moderate structural and functional divergence during evolution.

In eukaryotes, transcription factors are produced in the cytoplasm and perform their function in the nucleus to regulate the transcription of downstream genes. *PbGATA22* was a predicted bioinformatics analysis that also localizes in the nucleus (Figure 10) [36]. The coordination of numerous *cis*-acting elements regulates gene expression patterns [45]. Numerous hormone-responsive elements on the promoter regions of the *PbGATA* gene family were discovered in this work (Figure 12 and Table S6). The transcript levels of most potential *PbGATA* genes were stimulated to various degrees after post-treatment of fruit with exogenous hormones such as MeJA, SA, and ABA (as predicted). These findings are critical for pear fruit production in the field. By spraying various exogenous hormones onto pear fruits, we can control the metabolism of stone cells in the fruits and increase their quality [46]. Interestingly, none of the four putative *PbGATA* promoters had hormone-responsive elements, such as SA response elements in the *PbGATA* promoter regions. (Figure 12). This might be due to interactions between various types of plant hormones, which may encourage synergy and increases in each other’s content [47]. As a result, spraying hormones may cause a rise in the number of other hormones and increase peak gene expression in pear fruit. Gene expression patterns would provide important information about genes underlying activities [48,49]. Exogenous hormones (ABA) influence the expression levels of the GATA gene in Gossypium plants [46]. Previous research has shown that hormones on Chinese pear fruits can control the growth of stone cells and are involved in fruit ripening and senescence [50,51]. GATA TFs are involved in chloroplast biogenesis, hormones related to stress, light response, and floral development [17,36]. In *Arabidopsis thaliana*, *ATGATA2* is a positive regulator of phytohormones, which is significantly expressed in petioles and hypocotyls, and GATA24 [20,52] and *ATGATA28* were identified as two compulsory components of the cryptochrome1-mediated photoprotective response in *Arabidopsis Thaliana* [53]. In grapes, *VvGATA5, VvGATA2,* and *VvGATA7* were extremely expressed in flowers and *VvGATA5* was abundantly expressed in the berry ripening stage [16]. *SIGATA11* and *SIGATA12* in the root, while *SIGATA7* were expressed in root and flower, fruits, and *SIGATA25* gene involved in abs*cis*ic acid, flavonoids, and carotenoids [54]. GATA7 (*Cs5g26470*) was identified in citrus fruit which is involved in abscisic acid (ABA), glucose, and fructose [55]. *BnGATAs* showed a unique expression profile in various tissue of *Brassica napus* under abiotic stress [56].

In our study, qRT-PCR analysis was used to detect the expression profile of the GATA gene family in Chinese pear under three hormonal stress (ABA, SA, and MeJA) (Figure 11 and Figure 13). Several plants are improving their abiotic-biotic stress response after different exogenous hormonal treatments such as SA, MeJA, and ABA [57,58]. The fluctuations in various essential hormones such as SA, ABA, and MeJA occur as a response to stress [46]. We randomly selected 10 *PbGATA* genes from each subfamily to analyze their expression profile after the hormonal (MeJA, SA, and ABA) stress on pear fruit. In our study, all GATA genes revealed a strong upregulation under hormonal response, indicating that *PbGATA* members play a crucial role in the abiotic stress-related response.

## 4. Materials and Methods

### 4.1. Database Search and Physicochemical Characterization of GATA Genes

The whole-genome protein sequence, including a general function format file (GFF3) and CDS sequence of *Pyrus bretschneideri* was downloaded from the pear genome project (http://peargenome.njau.edu.cn/, accessed on 1 February 2021). The genome sequence of peach (*Prunus persica*), sweet cherry (*Prunus avium*), and Japanese apricot (*Prunus mume*) were downloaded from the genome database for Rosaceae (http://www.rosaceae.org/, accessed on 1 February 2021) and *Arabidopsis Thaliana* GATA protein sequences were obtained from TAIR website (https://www.arabidopsis.org/, accessed on 1 February 2021). Initially, the GATA family feature domain (GATA zinc finger) was obtained from the Pfam database [26]. Moreover, HMM search was conducted against the pear genome using the HMMER3 software package (E value: 0.001) [27]. The protein sequences of the resulting *PbGATA* TFs were retrieved and double-checked from a plant transcription factors database (http://planttfdb.cbi.pku.edu.cn/, accessed on 3 February 2021) [28]. Finally, the signature GATA domain for each protein was confirmed using SMART (http://smart.emblheidelberg.de, accessed on 5 February 2021) [29], Pfam (http://pfam.xfam.org, accessed on 5 February 2021) [30]. Additionally, NCBI web-based conserved domain search tool (https://www.ncbi.nlm.nih.gov/Structure/cdd/wrpsb.cgi, accessed on 8 February 2021) was used to verify the conserved domain. [59], and the protein sequences lacking the GATA domain were removed. Furthermore, the physical and chemical characteristics (amino acid lengths, isoelectric points, and molecular weights) and GO annotation analysis (molecular function, cellular component, biological process, and subcellular localization) were predicted using ExPASY online web server (https://web.expasy.org/compute_pi/, accessed on 13 March 2021) and CELLO v.2.5 with E-value 0.001 (http://cello.life.nctu.edu.tw/cello2go/alignment.php, accessed on 28 March 2021), respectively [60].

### 4.2. Chromosomal Localization and Introns/Exons Analysis

The starting and ending points of each GATA member were obtained from the Plant Transcription Factor Database website and confirmed by using the GFF3 file. Chromosomal localization was also validated by using CLC sequence viewer v7 [61]. Eventually, using MapChart v2.32, the GATA genes were scale-mapped on the chromosomes of *Pyrus bretschneideri*. Subsequently, the introns-exons alignment of *PbGATA* genes was analyzed by evaluating coding and genomic sequences using GSDS v2.0 (Gene Structure Display Server) (http://gsds.cbi.pku.edu.cn/, accessed on 4 April 2021) [62].

### 4.3. Collinearity Relationships, Mode of Gene Duplications, and Synonymous (ks) and Non-Synonymous (ka) Substitution

Multiple collinearity scan toolkit (MCScanX) was utilized for evaluating the mode of gene duplications (WGD, TD, PD, DSD, and TRD) event among, *P. persica*, *P. bretschneideri*, *P. mume* and *P. avium* with BLASTP by E-value set of 1 × 10^−5^ [63]. Collinearity analysis among *P. bretschneideri, P. persica, F. vesca, M. domestica, P. mume and P. avium* were recognized by MCScanX toolkits (https://github.com/wyp1125/MCScanX, accessed on 12 April 2021). The coding sequences of Paralogous gene pairs (PGPs) were aligned through ClustalW by using pairwise alignment in MAFFT online software (https://www.ebi.ac.uk/Tools/msa/mafft/, accessed on 13 April 2021). Finally, the alignment was added in DNA sequence polymorphism software (DnaSP v5.10.01) and after identifying the coding area, Nonsynonymous substitution rate (ka) and synonymous substitution rate (ks) were calculated by using ka/ks pipeline (https://github.com/qiaoxin/Scripts_for_GB/tree/master/calculate_Ka_Ks_pipeline, accessed on 23 April 2021) [64,65].

### 4.4. Conserved Motifs and Promoter Sequence Analysis

Moreover, the full-length sequence of amino acids was used in the MEME suite (Multiple EM for Motif Elicitation) (https://meme-suite.org/, accessed on 26 April 2021) online tools to classify conserved motifs and associated duplication events during the evolution of *PbGATA* members [32]. The default parameter configures rations were used for the corresponding exemptions. The maximal number of motifs to be identified was 20, and the possibility of motifs occurrence was varied per gene sequence. The graphical representation of the evolutionary relationship based on the described domains was developed. The promoter sequences (upstream of the ATG start codon with 1500 bp) of the *PbGATA* genes were carried out from the pear genome project and analyzed using online the PlantCARE database (http://bioinformatics.psb.ugent.be/webtools/plantcare, accessed on 2 May 2021).

### 4.5. In Silico Expression Analysis of Annotated PbGATA Genes

Transcriptomic data of *P. brestschneideri* was downloaded from the NCBI website (https://www.ncbi.nlm.nih.gov/sra, accessed on 10 May 2021) of d47ifferent fruit developmental stages with accession number SRX1595645, SRX1595648, SRX1595646, SRX1595647, SRX1595651, SRX1595650, SRX1595652. Meanwhile, RNA-seq reads of different organs (bud, leaves, stem, sepal ovary, and petal) were also downloaded with the following accession number SRR8119889, SRR8119895, SRR8119898, SRR8119903, SRR8119906, SRR8119907. Finally, FPKM (the fragments per kilobase of transcript per million mapped read) values were used for evaluating the expression patterns of *PbGATA* members and a heat map was visualized using R software.

### 4.6. Comparative Phylogenetic Analysis

ClustalX software was used to perform protein sequence-based on multiple sequence alignment for a comparison study of 32 *PbGATA* proteins from *Pyrus bretschneideri*, 18 proteins from *Prunus avium*, 22 from *Prunus persica*, 20 proteins from *Prunus mume*, and 30 from *Arabidopsis thaliana* and were aligned by using ClustalX tool (https://www.genome.jp/tools-bin/clustalx, accessed on 10 February 2021). The evolutionary relationship was computed using the Maximum likelihood method (ML-M) by using online IQ-tree software. Finally, all alignments were used to visualize a phylogenetic tree with itol software [66].

### 4.7. Plant Materials and Stress Treatment

Pear fruit samples were taken 39 days after flowering from a 45-year-old plant that was grown in a research horticulture garden in (Dangshan, Anhui) China. According to a previously mentioned procedure, 500 μM Methyl jasmonate (MeJA), 500 μM abscisic acid (ABA), and 200 μM salicylic acid (SA) were sprayed on the entire surface of the fruits [46,57] at 39 DAF. All fruit samples were obtained at 0 h, 1 h, 2 h, and 3 h. Finally, the fruits sample were instantly frozen in liquid nitrogen and stored at −80°C for further in vitro experiments.

### 4.8. Subcellular Localization of PbGATA Protein

To analyze subcellular localization, the *PbGATA22* gene containing stop codon was inserted into the pHB-35Spro eGFP vector with the primers in listed in (Table S2) [67]. p35S::*PbGATA22*::eGFP recombinant vector was transferred into Agrobacterium tumefaciens GV3101 competent cells (Shanghai, China, Weidi Biotechnology Company) and further recombinant vectors were transiently infiltered in Tobacco (*Nicotiana benthamiana*) epidermal cells. After cultivating in the greenhouse for 48 h without light, the agroinfiltered leaf area was detected using a LAS-AF confocal microscope (Leica, Wetzlar, Germany).

### 4.9. Isolation of Total RNA and Quantitative Real-Time PCR

To examine the qRT-PCR analysis, total RNA was extracted from frozen fruit tissue using RNAiso-mate Tissue Kit (Tiangen, Beijing, China). The purity and quantity of RNA were assessed by Nanodrop 1000 spectrophotometer (thermoscientic, Beijing, China). The RNA was extracted then reverse transcribed into the first-strand cDNA using a one-step RT-qPCR kit (Takara, Shanghai, China). As per the manufacturer instructions, quantitative RT-PCR (qRT-PCR) was performed using an SYBR green Premix Ex TaqTM kit (Takara) on an ABI 7500 real-time PCR detection system. The expression data were normalized with the tubulin gene as an internal control to investigate the gene expression level [68]. The sets of all primers used for qRT-PCR analysis were designed on Gen script online software (https://www.genscript.com/tools/, accessed on 4 June 2021) are listed in Supplementary Table S2. The experiments were conducted for three biological and technical replicates and relative expression levels for each gene were evaluated via the 2^−^^△△CT^ method [57,69].

## 5. Conclusions

In this study, a total of 92 GATA genes were scanned and isolated from four Rosaceae species (*Pyrus bretschneideri*, *Prunus avium*, *Prunus mume*, and *Prunus persica*) and classified into four subfamilies based on phylogeny. A systematic analysis of the GATA genes was carried out, including physicochemical characterization, conserved motif, chromosomal location, gene structure (introns/exons), evolutionary relationship, conserved domain, synonymous and non-synonymous ratios, transcriptomic, collinearity relationship, and *cis*-acting elements. Dispersed duplication (DSD) and whole-genome duplication (WGD) might highly contribute to the expansion of GATA genes. In addition, qRT-PCR results revealed that *PbGATAs* had a significant role related to abiotic stress. Subcellular localization of *PbGATA22* by transient expression of GFP fusion protein in tobacco cells predicted that the majority of GATA family proteins are localized in the nucleus of leaf panels. These results provide basic information that may facilitate the evolutionary relationship, molecular mechanism, and functional analysis of *PbGATA* genes to understand their roles in pear fruits.

## Data Availability

The whole-genome protein sequence of Chinese pear (*Pyrus bretschneideri*) was downloaded from the pear genome project (http://peargenome.njau.edu.cn/, accessed on 28 September 2021). The genome sequence of Prunus persica, Prunus avium and Prunus mume were downloaded from the genome database for Rosaceae (http://www.rosaceae.org/, accessed on 28 September 2021) and Arabidopsis GATA protein sequences were obtained from TAIR website (https://www.arabidopsis.org/, accessed on 28 September 2021). Transcriptomic data of *P. brestschneideri* was downloaded from the NCBI website (https://www.ncbi.nlm.nih.gov/sra, accessed on 28 September 2021) of different fruit developmental stages with accession number SRX1595645, SRX1595648, SRX1595646, SRX1595647, SRX1595651, SRX1595650, SRX1595652. Meanwhile, RNA-seq reads of different organs (bud, leaves, stem, sepal ovary and petal) were also downloaded with following accession number SRR8119889, SRR8119895, SRR8119898, SRR8119903, SRR8119906, and SRR8119907.

## Supplementary Materials

The following are available online at https://www.mdpi.com/article/10.3390/ijms222212492/s1.
